# Supplementary material for: Gene panel selection for targeted spatial transcriptomics
Source: Genome Biol. 2024 Jan 25;25:35. doi: 10.1186/s13059-024-03174-1 (PMC10811939; doi:10.1186/s13059-024-03174-1)
Supplement: Supplementary file 1 — Additional file 1: Figure S1. Schematic of the Bayesian model for platform effect estimation. Figure S2. Bayesian model captures platform effect between scRNA-seq and targeted spatial transcriptomics technologies. Figure S3. Impact of platform effect on the spatial transcriptomics measurement simulation and cell type classification. Figure S4. Impact of multiplicative platform effect on cell type classification and average cell depth of the simulated spatial transcriptomics data for the Codeluppi dataset. Figure S5. Comparison between gpsFISH and other gene selection methods on the Zhang and Codeluppi dataset. Figure S6. Comparison between gpsFISH and other gene selection methods using random forest as classifier. Figure S7. High redundancy across optimizations using gpsFISH. Figure S8. Weighted gene panel selection based on probe count per gene. Figure S9. Gene panel selection with ligand activity as gene weight. Figure S10. Gene panel selection with cell type hierarchy on the Tietscher dataset. Figure S11. Accuracy of optimized gene panels using flat vs. hierarchical gene selection. Supplementary texts. [file 13059_2024_3174_MOESM1_ESM.docx]

**
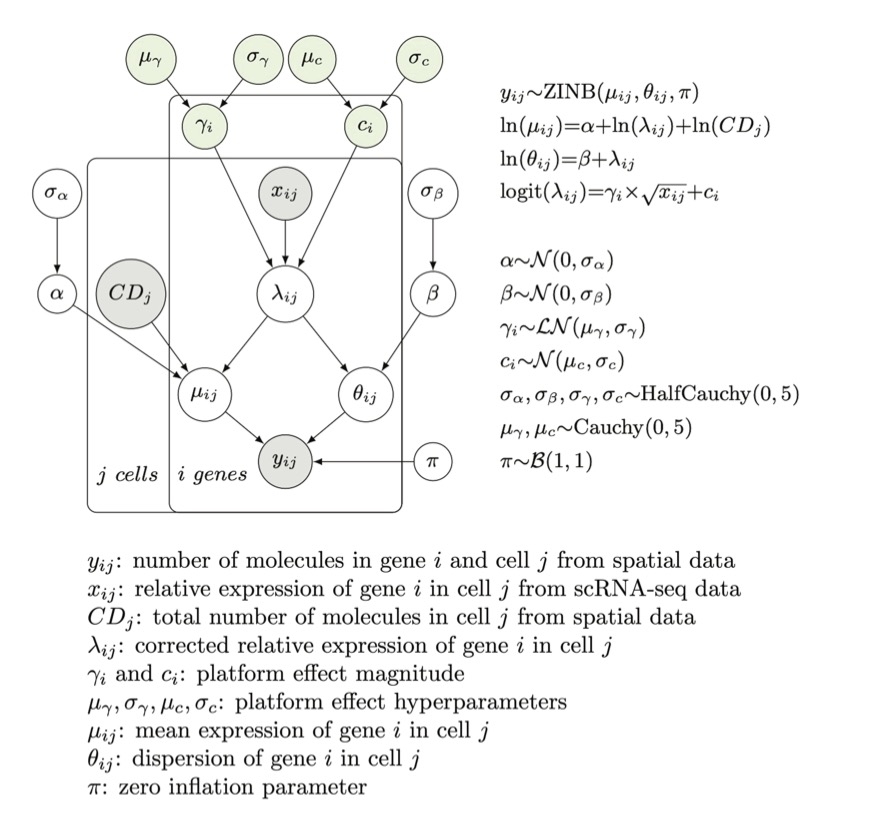
**

**Figure S1**: Schematic of the Bayesian model for platform effect estimation.

Circles in gray represent observed variables. Circles in green correspond to platform effect related variables to be estimated.

**
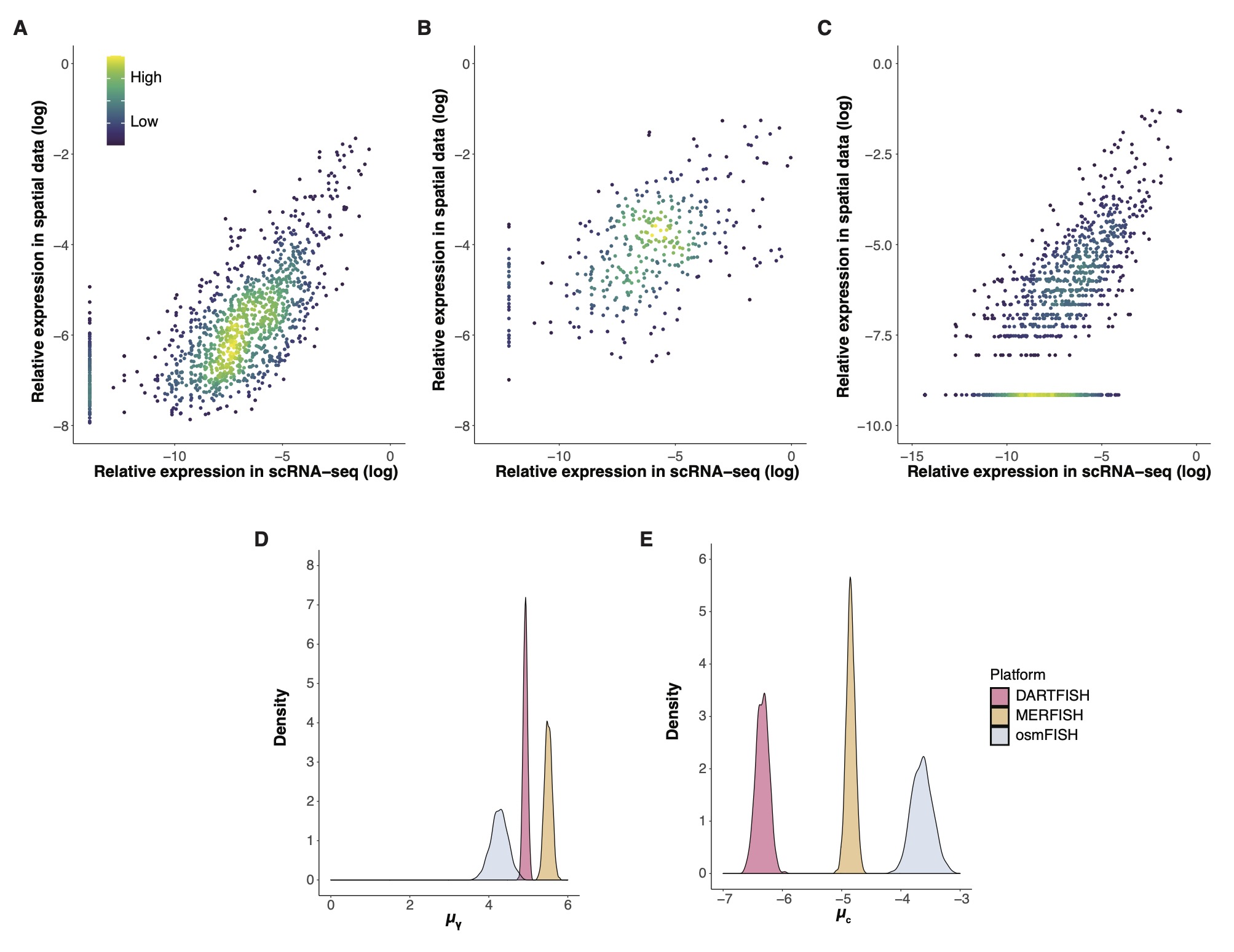
**

**Figure S2:** Bayesian model captures platform effect between scRNA-seq and targeted spatial transcriptomics technologies.

**A-C:**

Scatter plot showing the log transformed relative expression of genes measured by scRNA-seq vs. simulated spatial transcriptomics data using fitted Bayesian model for Moffit (**A**), Codeluppi (**B**), and Zhang (**C**), respectively.

**D-E:**

Density plot showing the estimated posterior distribution of $\mu_{\gamma}$ (**D**) and $\mu_{c}$. (**E**).

**
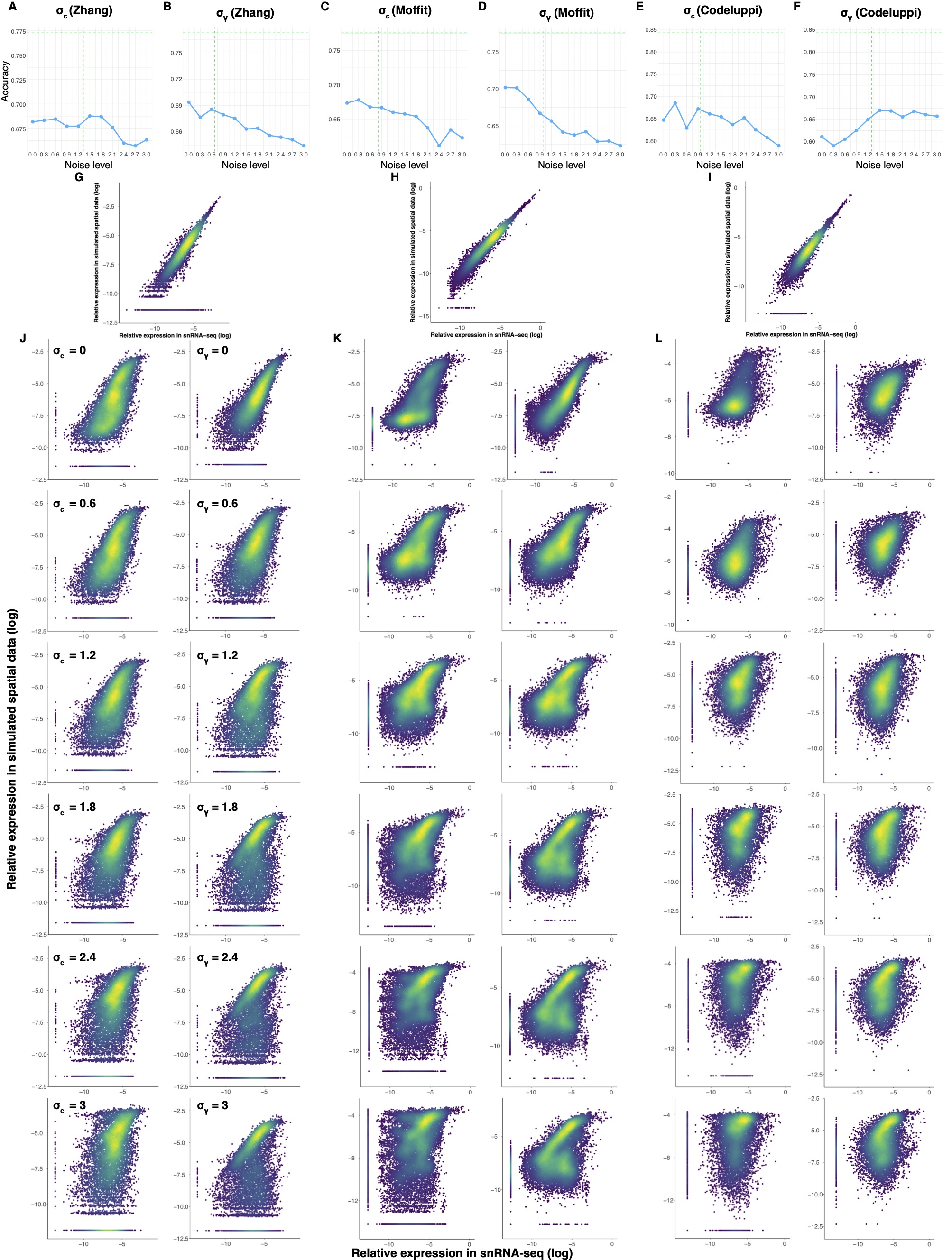
**

**Figure S3**: Impact of platform effect on the spatial transcriptomics measurement simulation and cell type classification.

**A-F:**

Classification accuracy at different levels of additive ($\sigma_{c}$) and multiplicative ($\sigma_{\gamma}$) platform effect for the Zhang (**A, B**), Moffit (**C, D**), and Codeluppi (**E, F**) dataset. The horizontal dashed line corresponds to the classification accuracy based on simulated spatial transcriptomics measurement without platform effect. The vertical dashed line corresponds to the mean of the posterior distribution of $\sigma_{c}$ or $\sigma_{\gamma}$ estimated from the Zhang, Moffit, and Codeluppi dataset.

**G-I:**

Scatter plot showing the log transformed relative expression of genes measured by both scRNA-seq and simulated spatial transcriptomics without platform effect across three datasets, Zhang (**G**), Moffit (**H**), and Codeluppi (**I**), respectively. A small value is added to avoid negative infinity after log transformation. Each dot represents the relative expression of one gene in one cell type. Denominator for relative expression calculation is from genes in the optimized gene panel at level 2 cell type annotation of each dataset. Color indicates density of dots.

**J-L**:

Scatter plot showing the log transformed relative expression of genes measured by both scRNA-seq and simulated spatial transcriptomics at different levels of additive ($\sigma_{c}$) and multiplicative ($\sigma_{\gamma}$) platform effect across three datasets, Zhang (**J**), Moffit (**K**), and Codeluppi (**L**), respectively.

**
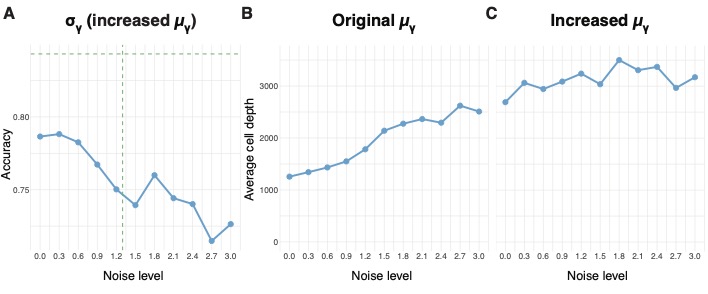
**

**Figure S4**: Impact of multiplicative platform effect on cell type classification and average cell depth of the simulated spatial transcriptomics data for the Codeluppi dataset.

**A:**

Classification accuracy at different levels of multiplicative ($\sigma_{\gamma}$) platform effect for the Codeluppi dataset after increasing the level of $\mu_{\gamma}$. The horizontal dashed line corresponds to the classification accuracy based on simulated spatial transcriptomics measurement without platform effect. The vertical dashed line corresponds to the mean of the posterior distribution of $\sigma_{\gamma}$ estimated from the Codeluppi dataset.

**B-C:**

Average cell depth of the simulated spatial transcriptomics data at different levels of multiplicative ($\sigma_{\gamma}$) platform effect for the Codeluppi dataset before (**B**) and after (**C**) increasing the level of $\mu_{\gamma}$.

**
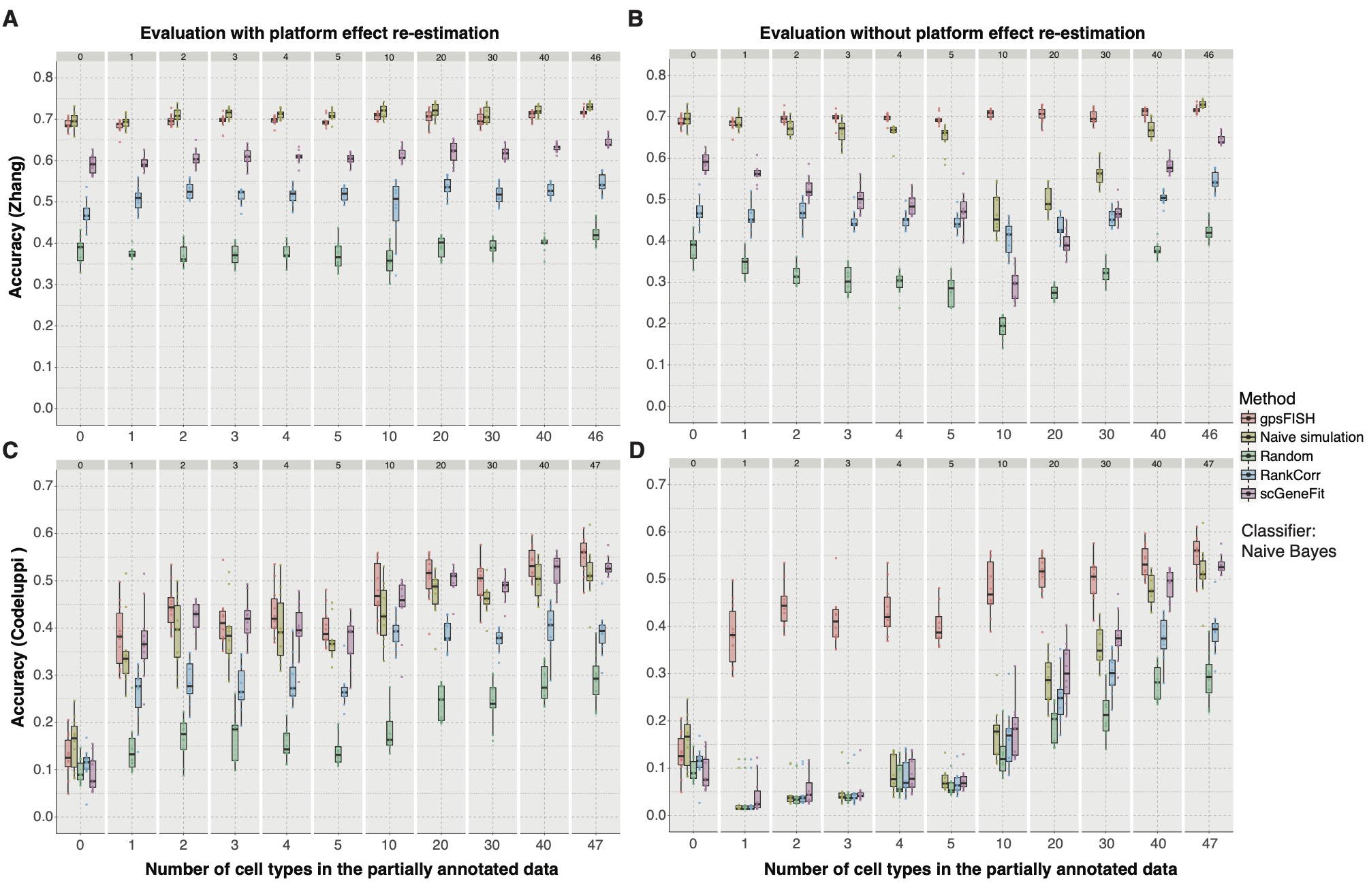
**

**Figure S5:** Comparison between gpsFISH and other gene selection methods on the Zhang and Codeluppi dataset.

Box plot showing classification accuracy distribution of gene panels selected by 5 gene panel selection methods at different levels of partial annotation. (**A**) Zhang dataset using evaluation with platform effect re-estimation. (**B**) Zhang dataset using evaluation without platform effect re-estimation. (**C**) Codeluppi dataset using evaluation with platform effect re-estimation. (**D**) Codeluppi dataset using evaluation without platform effect re-estimation. Naïve Bayes is used as classifier.

**
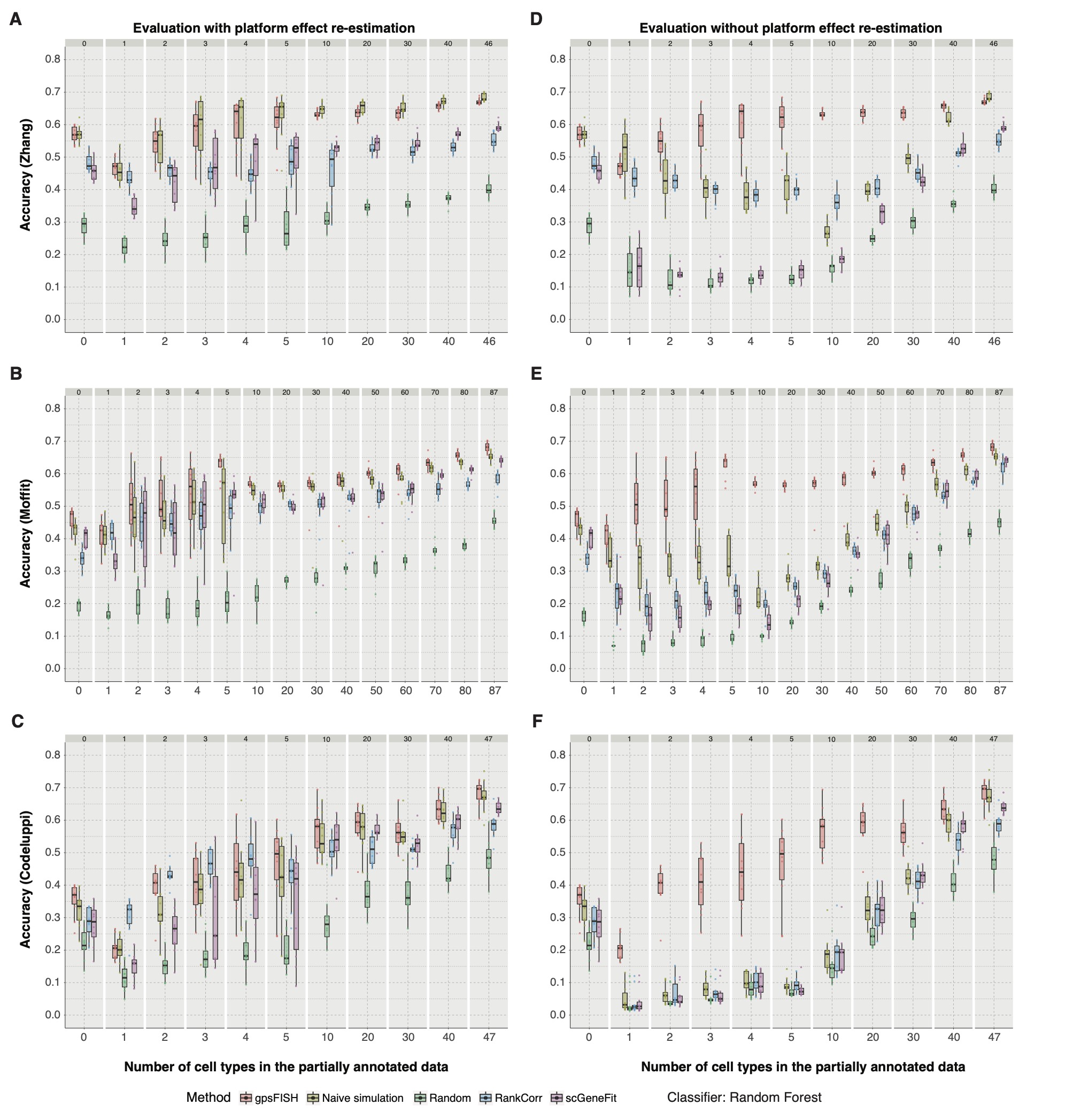
**

**Figure S6:** Comparison between gpsFISH and other gene selection methods using random forest as classifier.

Box plot showing classification accuracy distribution of gene panels selected by 5 gene panel selection methods at different levels of partial annotation for the three datasets using evaluation with (**A-C**) and without (**D-E**) platform effect re-estimation. Random forest is used as classifier.

**
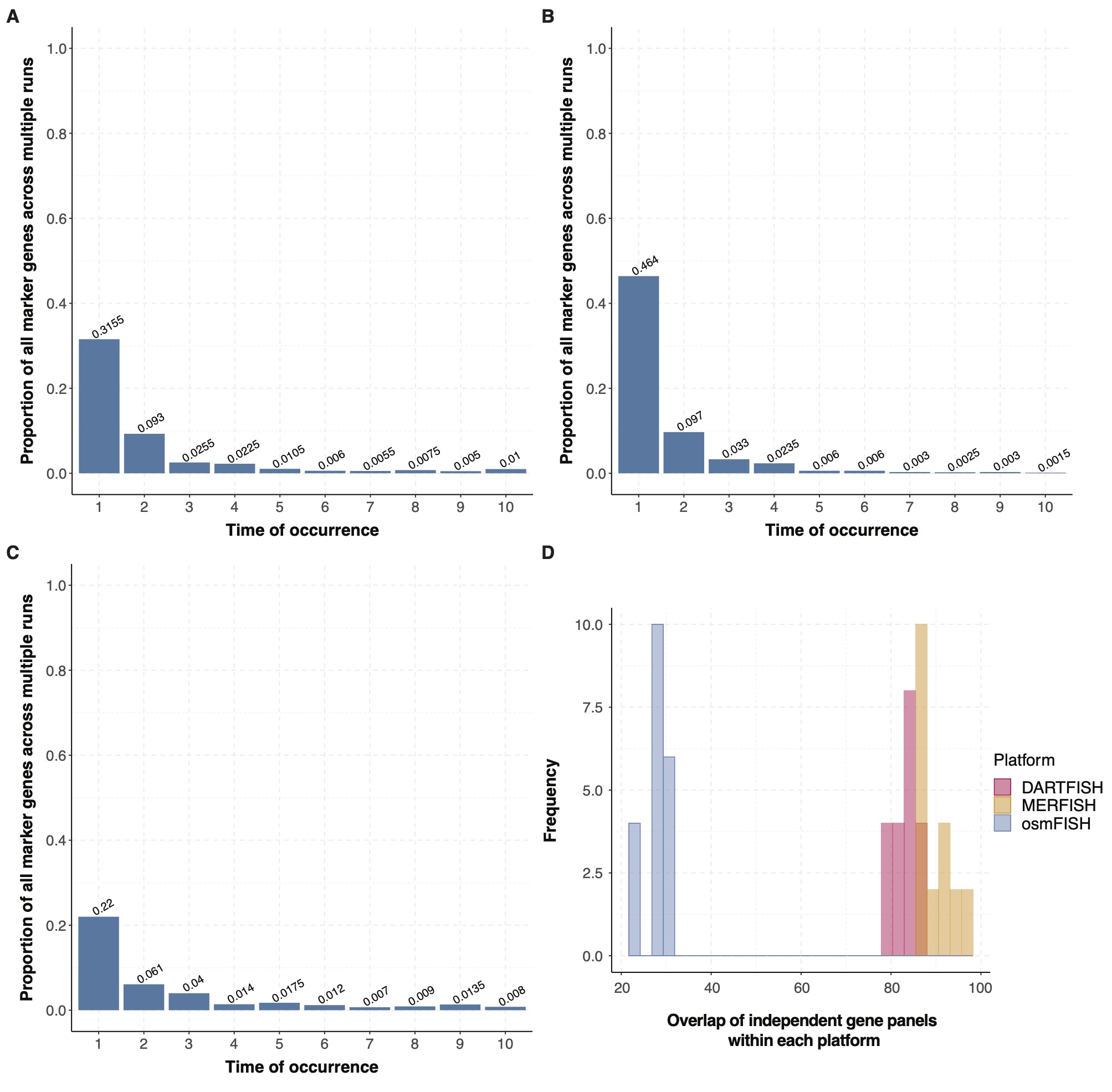
**

**Figure S7:** High redundancy across optimizations using gpsFISH.

**A-C:**

Bar plot showing among all the genes selected in 10 optimizations, the percentage of them that are included in 1 to 10 optimized panels for Moffit (**A**), Codeluppi (**B**), and Zhang (**C**) dataset at level 1 cell type annotation.

**D:**

Distribution of overlap of independent gene panels across 10 optimizations within each platform at level 2 cell type annotation.

**
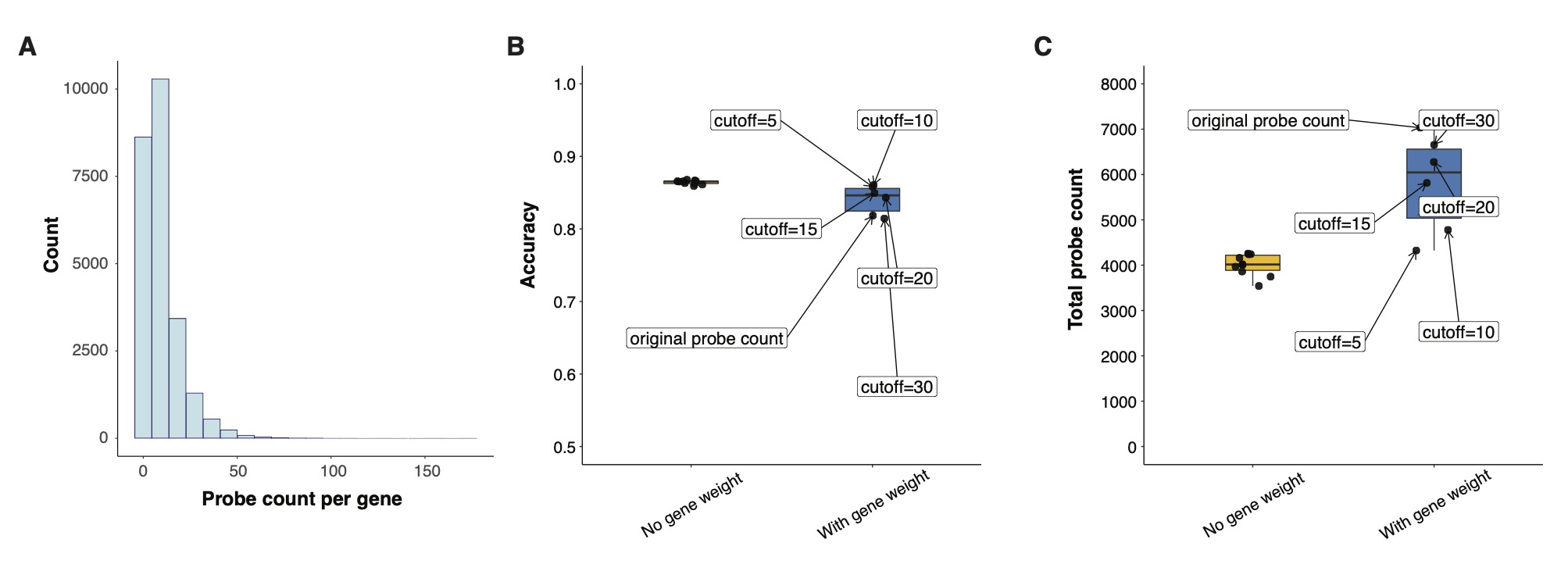
**

**Figure S8:** Weighted gene panel selection based on probe count per gene.

**A:**

Distribution of probe count per gene for the Zhang dataset.

**B-C:**

Distribution of accuracy (**B**) and total number of probes (**C**) of optimized gene panels from optimization without and with gene weight. Optimization without gene weight is performed 10 times. Optimization with gene weight is performed 6 times, each time with a different probe count cutoff (no cutoff, 5, 10, 15, 20, 30).

**
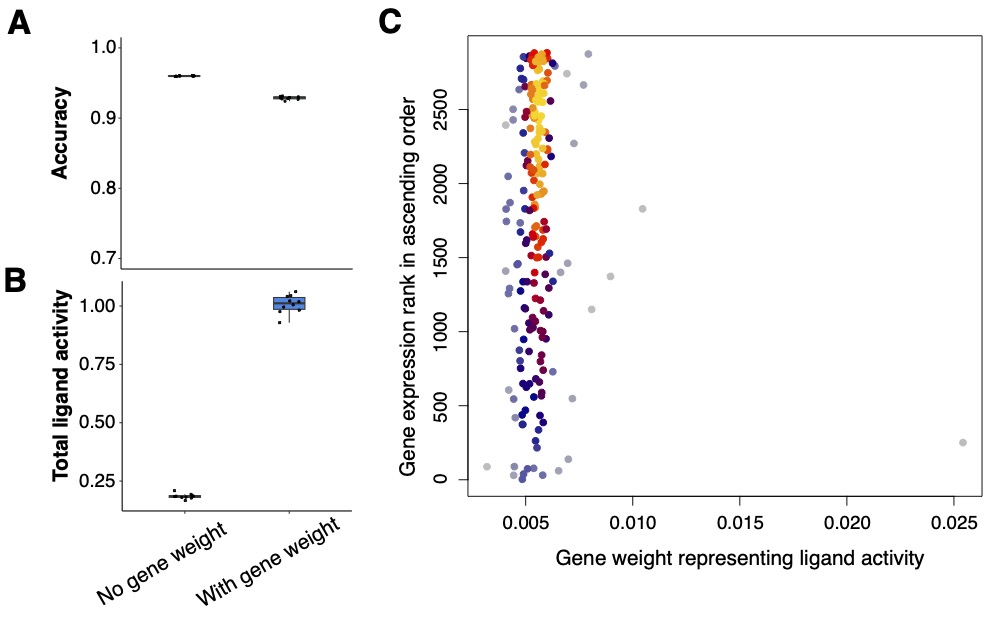
**

**Figure S9:** Gene panel selection with ligand activity as gene weight.

**A:**

Accuracy of optimized gene panels without vs. with gene weight across 10 optimizations.

**B:**

Total ligand activity of optimized gene panels without vs. with gene weight across 10 optimizations.

**C:**

Scatterplot showing the relationship between gene weight and gene expression of genes in the 10 optimized gene panels with gene weight. Each dot represents a gene with non-zero ligand activity in the 10 optimized gene panels with gene weight. Genes with higher ligand activity will have higher gene weight. Genes with higher expression will have larger rank. Color indicates density of dots.

**
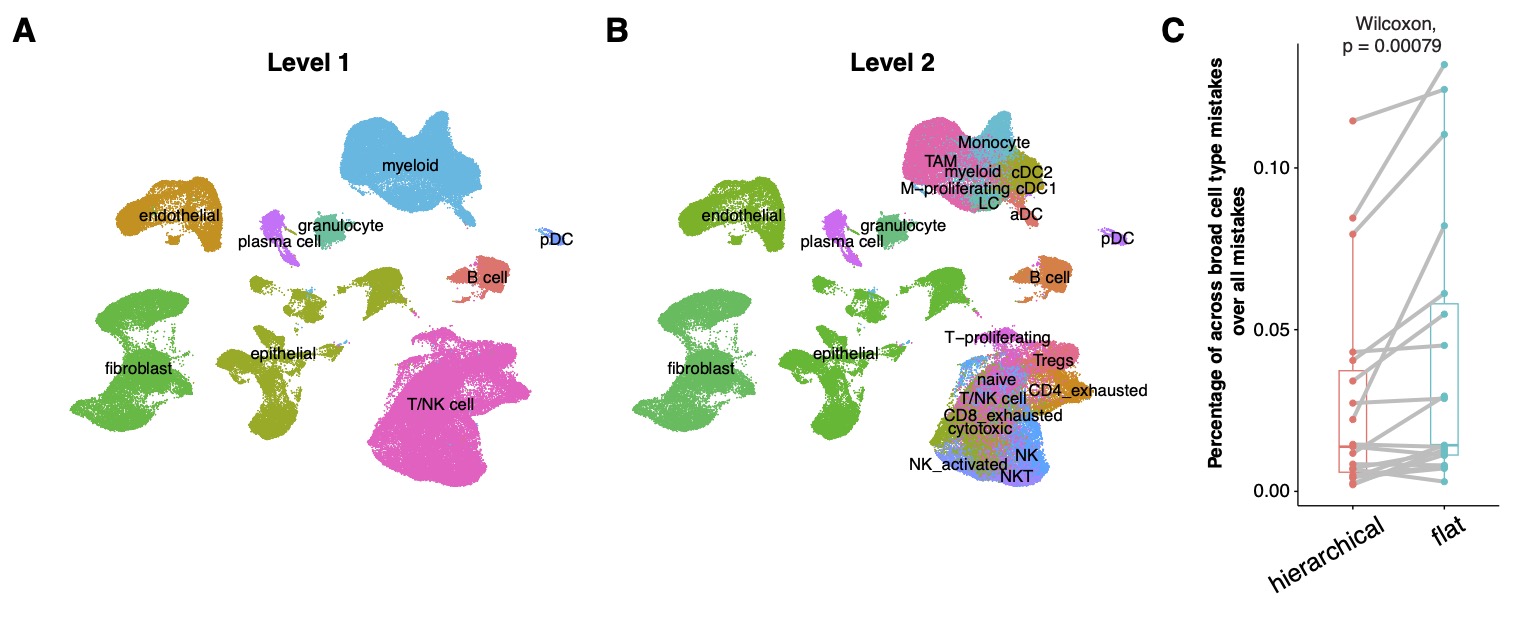
**

**Figure S10:** Gene panel selection with cell type hierarchy on the Tietscher dataset.

**A-B:**

UMAP of cells based on the Tietscher dataset at level 1 (**A**) and 2 (**B**) cell type annotation.

**C:**

Percentage of across broad cell type (level 1) misclassifications over all misclassifications for flat vs. hierarchical classification on the Tietscher dataset. Each dot represents one cell type with dots representing the same cell type connected. Wilcoxon paired test is performed between the percentages from flat vs. hierarchical classification and the p value is shown.

**
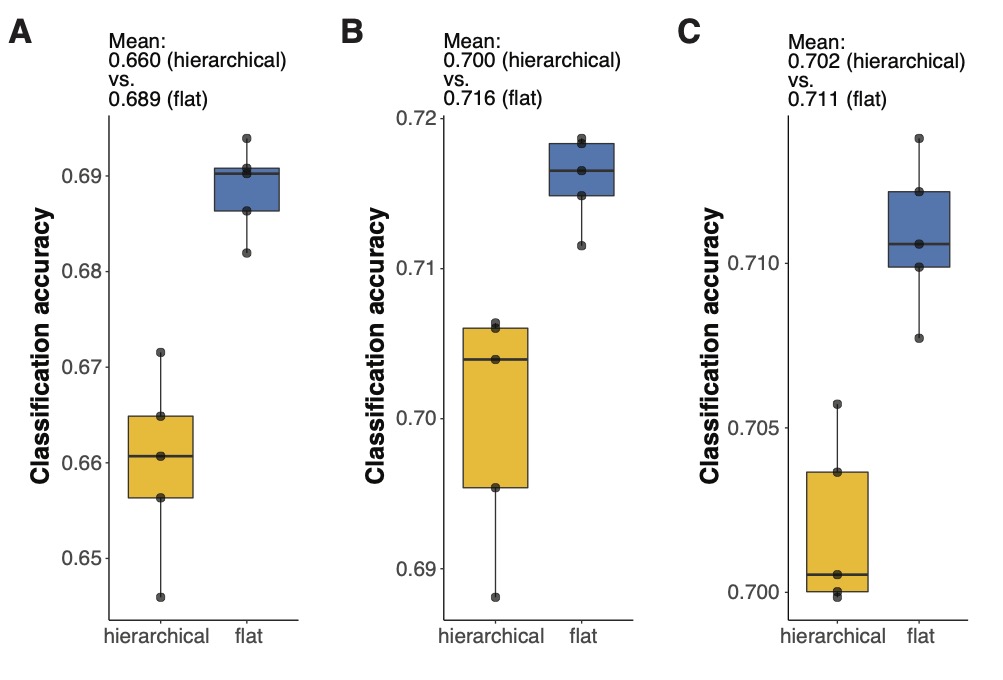
**

**Figure S11:** Accuracy of optimized gene panels using flat vs. hierarchical gene selection.

**A-C:**

Distribution of accuracy of optimized gene panels using flat vs. hierarchical gene selection for Moffit (**A**), Codeluppi (**B**), and Zhang (**C**), respectively. Both flat and hierarchical gene selection are performed 5 times.
