# Supplementary material for: Gene panel selection for targeted spatial transcriptomics
Source: Genome Biol. 2024 Jan 25;25:35. doi: 10.1186/s13059-024-03174-1 (PMC10811939; doi:10.1186/s13059-024-03174-1)
Supplement: Supplementary file 4 — Additional file 4. Peer review history. [file 13059_2024_3174_MOESM4_ESM.docx]

**Review history**

**First round of review**

**Reviewer 1**

Please consider the following questions:

- Are the methods appropriate to the aims of the study, are they well described, and are necessary controls included?

The authors described a gene selection platform for spatial transcriptomics using scRNAseq data, Bayesian modeling of expression data, and genetic algorithms.

- Are the conclusions adequately supported by the data shown?

Three paired scRNA-seq and targeted spatial transcriptomics datasets were included in the testing, validation, and demonstrations. sufficient for technical validations and comparisons.

- Are sufficient details provided to allow replication and comparison with related analyses that may have been performed?

GitHub was included with clear instructions. Demonstrations with alternative gene selections are also interesting.

- Is the method likely to be of broad utility? Is any software component easy to install and use? Please indicate briefly the novel features and/or advantages of the method, and/or please reference the relevant publications and which methods, if any, it should be compared with.

Yes, gene selection for targeted spatial transcriptomics is an important issue and this package can be broadly used.

- Is the paper of broad interest to others in the field, or of outstanding interest to a broad audience of biologists?

Yes, brain and kidney datasets were used for demonstrations. An extension of this to other applications in immunology, cancer biology, etc. would even be more interesting.

While the article is technically sound, there a few major concerns:

1) Can authors include spatial statistics such as genes controlling cell-to-cell interactions in their pipeline.

It appears that the gene optimizations are mostly based on expression differences and distributions, but the spatial distributions are not systematically included.

For instance, if low expressed genes control crucial cell communication, how would this pipeline capture these genes in their target list?

Also, if there is specific gene group that the users are interested, is there any way to include them in addition to the target genes identified by gpsFISH.

2) Can authors apply gpsFISH to immunology for specifically studying spatial transcriptional regulation in immune responses of tissues? Other datasets would be also interesting beyond brain.

3) The simulated data - would that effect the result of genetic algorithm? Authors should define clear boundary conditions of how technical noise is included in simulations and how they affect the downstream analysis?

**Reviewer 2**

The problems/objectives of the study are well defined. The solution is convincing. The writing is fairly good. I have no further comments.

**Authors’ response to reviewers**

Response to Reviewers

Reviewer #1:
- Are the methods appropriate to the aims of the study, are they well described, and are necessary controls included?
The authors described a gene selection platform for spatial transcriptomics using scRNAseq data, Bayesian modeling of expression data, and genetic algorithms.

- Are the conclusions adequately supported by the data shown?
Three paired scRNA-seq and targeted spatial transcriptomics datasets were included in the testing, validation, and demonstrations. sufficient for technical validations and comparisons.

- Are sufficient details provided to allow replication and comparison with related analyses that may have been performed?
GitHub was included with clear instructions. Demonstrations with alternative gene selections are also interesting.

- Is the method likely to be of broad utility? Is any software component easy to install and use? Please indicate briefly the novel features and/or advantages of the method, and/or please reference the relevant publications and which methods, if any, it should be compared with.
Yes, gene selection for targeted spatial transcriptomics is an important issue and this package can be broadly used.

- Is the paper of broad interest to others in the field, or of outstanding interest to a broad audience of biologists?
Yes, brain and kidney datasets were used for demonstrations. An extension of this to other applications in immunology, cancer biology, etc. would even be more interesting.

We thank the Reviewer for the positive comments.

While the article is technically sound, there a few major concerns:

1) Can authors include spatial statistics such as genes controlling cell-to-cell interactions in their pipeline.
It appears that the gene optimizations are mostly based on expression differences and distributions, but the spatial distributions are not systematically included.
For instance, if low expressed genes control crucial cell communication, how would this pipeline capture these genes in their target list?
Also, if there is specific gene group that the users are interested, is there any way to include them in addition to the target genes identified by gpsFISH.

We fully appreciate Reviewer’s point that target gene selection often needs to incorporate requirements and preferences that go beyond gene expression specificity. Such preferences may be based on various outside data sources, including existing spatial experiments. gpsFISH allows users to incorporate any such secondary preferences during gene panel optimization by specifying custom gene weights (line 308287). Gene weight is a value between 0 and 1, and higher weight genes are more likely to be selected for the panel (lines 754 723 - 757726).

Following Reviewer’s suggestion, we have revised the manuscript to demonstrate how gene weights can be used to generate panels that will be informative cell-to-cell interactions, even if many ligands are expressed at lower levels. In this new example (page 14), we used the Moffit et al. dataset to calculate putative activity score for each ligand using scRNA-seq data. This ligand activity was then used as gene weight during panel selection. Performing the gene panel selection 10 times with and without gene weight showed that the optimizations with gene weight achieved a significantly higher overall ligand activity coverage with only minor decrease of classification accuracy. In addition, the final optimized gene panels include genes with high ligand activity but relatively low gene expression compared to other candidate genes. These results demonstrated that gpsFISH can incorporate secondary preferences beyond gene expression specificity such as genes controlling cell-to-cell interactions into the optimization through gene weight. Moreover, gpsFISH is able to capture genes with high gene weight (in this case, high ligand activity) but low expression. The revised manuscript now describes the analysis and results of such optimizations (Analysis: line 903 869 to 909875. Result: line 334 307 to 348320).

Of note, we did not implement a specific mechanism to account for spatial statistics, as at the stage of gene panel designs it is rare for the users to have spatial data that would include the ligands of interest in the desired experimental condition. However, the general gene weight mechanism in gpsFISH would allow potential results of spatial analysis to be incorporated in the same manner. In addition to this soft biasing, gpsFISH also allows to specify sets of pre-selected genes to be incorporated into the panels (i.e. as a hard requirement, see lines 302 281 to 306 285 and 769 738 to 771740).


2) Can authors apply gpsFISH to immunology for specifically studying spatial transcriptional regulation in immune responses of tissues? Other datasets would be also interesting beyond brain.

We thank the Reviewer for this suggestion. To apply gpsFISH to immunology for studying the spatial distribution of gene expression, we ran gpsFISH optimization on a scRNA-seq dataset collected from human breast cancer samples (Tietscher et al.). This dataset provides a comprehensive single-cell map of T cell exhaustion-associated immune environments in human breast cancer. By performing gene selection using the hierarchical classification scheme, we show that gpsFISH provides a flexible framework to account for detailed cell type hierarchies. Description of the data, analyses, and results have been added to the manuscript (Data: lines 603 572 to 616585. Analyses: lines 820 to 824. Result: lines 372 352 to 395364).


3) The simulated data - would that effect the result of genetic algorithm? Authors should define clear boundary conditions of how technical noise is included in simulations and how they affect the downstream analysis?

We thank the Reviewer for the suggestion. To demonstrate how the level of platform effect affect the spatial transcriptomics data simulation and cell type classification, we performed spatial transcriptomics data simulation and cell type classification at different levels of additive and multiplicative platform effect on the three datasets. For spatial transcriptomics measurement simulation, we observed a poorer agreement between the scRNA-seq data and the simulated spatial transcriptomics data at higher level of additive and multiplicative platform effects. For cell type classification, we observed that higher level of additive platform effect (σ_c) reduces cell type classification accuracy. Higher level of multiplicative platform effect (σ_γ) reduces cell type classification accuracy when μ_γ was sufficiently high. When μ_γ is low, increasing level of multiplicative platform effect will first lead to an increase cell type classification and then saturate, due to increase in the overall cell depth. These analysis and the results have been added to the manuscript (Analysis: lines 773742-787757. Results: lines 190-225205).


Reviewer #2: The problems/objectives of the study are well defined. The solution is convincing. The writing is fairly good. I have no further comments.

We thank the Reviewer for their positive assessment of our paper.
